# Extracellular vesicles metabolic changes reveals plasma signature in stage-dependent diabetic kidney disease

**DOI:** 10.1080/0886022X.2022.2118067

**Published:** 2022-11-11

**Authors:** Youjin Pan, Hui Yang, Tucan Chen, Jian Jin, Luya Ruan, Liang Hu, Li Chen

**Affiliations:** a Department of Endocrinology, Qilu Hospital, Cheeloo College of Medicine, Shandong University, Jinan, China; b Department of Endocrinology, The Second Affiliated Hospital and Yuying Children’s Hospital of Wenzhou Medical University, Wenzhou, China; c Institute of Endocrine and Metabolic Diseases of Shandong University, Jinan, China; d Key Laboratory of Endocrine and Metabolic Diseases, Shandong Province Medicine & Health, Qingdao, China; e Jinan Clinical Research Center for Endocrine and Metabolic Diseases, Jinan City, P. R. China; f Department of Ophthalmology, The Second Affiliated Hospital and Yuying Children’s Hospital of Wenzhou Medical University, Wenzhou, China; g School of Ophthalmology & Optometry, School of Biomedical Engineering, Wenzhou Medical University, Wenzhou, China

**Keywords:** Extracellular vesicle, exosomes, diabetic kidney disease, diabetes mellitus

## Abstract

**Objective:**

Early diagnosis of diabetic kidney disease (DKD) has long been a complex problem. This study aimed to analyze the metabolomic characteristics of plasma extracellular vesicles (EVs) at different stages of DKD in order to evaluate the metabolites of plasma EVs and select new biomarkers for the early diagnosis of DKD.

**Patients and methods:**

A total of 78 plasma samples were collected, including samples from 20 healthy controls, 20 patients with type 2 diabetes mellitus (T2DM), 18 patients with DKD stage III, and 20 patients with DKD stage IV. In addition, EVs were isolated for metabolomics analysis.

**Results:**

The results identified differences in EV metabolomic characteristics in DKD patients at different stages, as well as significant differences in EV metabolomics between T2DM patients without DKD and patients with DKD. Ten Significantly differential metabolites were associated with the occurrence and progression of DKD. Uracil, LPC(O-18:1/0:0), sphingosine 1-phosphate, and 4-acetamidobutyric acid were identified as potential early biomarkers for DKD, showing excellent predictive performance.

**Conclusion:**

Uracil, LPC(O-18:1/0:0), sphingosine 1-phosphate, and 4-acetamidobutyric acid exhibited potential as suitable biomarkers for early DKD diagnosis. Unexpectedly, combining these four candidate metabolites resulted in enhanced predictive ability for DKD.

## Introduction

Diabetic kidney disease (DKD) results from renal damage caused by diabetes mellitus (DM) and may involve the whole kidney (including glomeruli, tubules, interstitium, and vessels) [1]. Clinically, it is mainly characterized by persistent albuminuria and/or progressive decline of estimated glomerular filtration rate (eGFR). Features include urinary albumin to creatinine ratios (UACR) ≥30 mg/g (or ≥3 mg/mmol) and/or eGFR <60 mL/min/1.73 m^2^ lasting for more than three months. Eventually, DKD develops into end-stage kindey disease (ESKD) [2]. Notably, more than 20% of Type 2 DM (T2DM) patients already have DKD when they are diagnosed with diabetes [3].

Urinary albumin excretion is not always a reliable biomarker as it can be affected by a variety of physiological or pathological factors that may lead to false-positive results; these include exercising within 24 h, infection, fever, chronic cardiac failure, hyperglycemia, menstrual periods, blood pressure, and ketoacidosis. Increased urinary albumin excretion has been shown to be associated with endothelial cell dysfunction [4]. On the other hand, cumulative studies have shown the existence of a nonproteinuric DKD phenotype [5–7]. In clinical practice, some T2DM patients with DKD exhibit decreased GFR without proteinuria, and many develop ESKD alongside a progressive decline in GFR without significant albuminuria. In addition, recent studies on renal biopsies suggest that there may be differences in the renal morphologic lesions caused by albuminuric and nonalbuminuric forms of DKD [8,9].

The above evidence illustrates the complexity of DKD as well as the difficulty of early diagnosis, highlighting the urgency of identifying highly reliable early biomarkers. Extracellular vesicles (EVs), particularly small EVs (exosomes), carry a variety of compounds such as proteins, nucleic acids, and metabolites from source cells, and mediate cell-to-cell interactions, making them a current research hotspot due to their potential diagnostic value. At present, many studies indicate that biological markers derived from exosomes may become important targets and markers for early DKD diagnosis, including gelatinase, ceruloplasmin, DPP4, Elf3, miRNA-320C, and WT1 mRNA [10–15]. Furthermore, some researchers have found that plasma exosomes may aid in the accurate staging and treatment of DKD. For example, miRNA-126 within EVs may predict DKD pathogenesis and serve as a marker of therapeutic intervention effects [16,17] owing to the regulatory role of miRNA-126 in the response to VEGF (an essential factor in the pathogenesis of DKD). Another miRNA involved in DM and its complications, miRNA-29, was reported to play a protective role in fibrotic disease (including kidney fibrosis [18]) and to be involved in DKD pathogenesis in DM mice [19,20].

As a metabolic disease, T2DM is characterized by a variety of metabolic phenotypes, such as hyperglycemia, dyslipidemia, and obesity. In addition, metabolic factors and hemodynamic factors are involved in the pathogenesis of DKD, leading to alterations in intracellular signaling molecules and enhanced secretion of growth factors and cytokines. Therefore, exosome metabolomics may reveal early biomarkers of DKD and provide new insights into mechanisms and treatments. To date, we have not identified other studies carrying out plasma exosome metabolomics analysis in patients with DKD. This study aimed to explore potential biomarkers for the staging and early diagnosis of DKD by analyzing metabolites found in EVs.

## Material and methods

### Research subjects

Participants were recruited between 1 December 2020, and 30 September 2021, and informed consent was obtained according to the scheme approved by the Second Affiliated Hospital and Yuying Children’s Hospital of Wenzhou Medical University. An endocrinologist diagnosed the clinical stage classification of DKD. T2DM was confirmed according to the American Diabetes Association’s 2021 standards [21]. Normal UACR is defined as <30 mg/g (or 3 mg/mmol), microalbuminuria (DKD stage III) is defined as 30 mg/g (or 3 mg/mmol) ≤ UACR <300 mg/g (or 30 mg/mmol), and macroalbuminuria (DKD stage IV) is defined as UACR ≥300 mg/g (or 30 mg/mmol). The T2DM patients were aged between 30 and 70 years old and lacked acute diabetic complications such as ketoacidosis, hypertonic hyperglycemia, and hypoglycemia. The exclusion criteria included Type 1 DM, inflammatory diseases, cancer, and severe liver, kidney, or heart disease. After recruitment, participants were divided into four groups: healthy controls (*n* = 20), T2DM group (*n* = 20), DKD III group (*n* = 18), and DKD IV group (*n* = 20).

Blood samples were collected in EDTA-coated tubes After centrifugation at 4,200 × g for 15 min to remove hematocytes, the plasma was filtered through a 0.22 μm PVDF membrane filter and frozen at −80 °C for later use.

This study was approved by the Ethics Committee of the Second Affiliated Hospital of Wenzhou Medical University (No. 2021-K-329-02). The procedures were in accordance with the Helsinki Declaration.

### Isolation of EVs using TiO_2_ microparticles

EVs were isolated using TiO_2_ microparticles, as in previous reports [22]. Briefly, plasma samples were incubated with beads at 4 °C for 5–10 min. The beads were then collected using a magnet and washed using phosphate buffer saline (PBS). After treatment in alkaline solution (10% NH_3_ in H_2_O) and ultrafiltration (Millipore, 30 kDa), the final EV supernatant was resuspended in 200 μL PBS.

Transmission electron microscope (TEM) imaging was used to measure the size of EVs. After being fixed onto carbon grids using 4% PFA and washed in PBS, the EVs were then incubated with 50 μL 1% glutaraldehyde solution and washed with distilled water. After staining with 2% uranyl acetate for 30 s, EVs were imaged using TEM. Western blotting was used to identify characteristic proteins. Protein quantity was measured using a Qubit^TM^ Protein Assay Kit. Primary antibodies, including anti-CD63, anti-CD9 and anti-TSG101, were used at a dilution of 1:1000. The blotting results were imaged by enhanced chemiluminescence for immunodetection.

### Metabolic compound profiling

The EV samples were first mixed with 1 mL of pre-cooled 70% methanol. The mixture was then frozen in liquid nitrogen for 5 min and thawed on ice for 5 min (repeated three times). After sonication at 30 Hz for 3 min and centrifugation at 12,000 rpm for 10 min at 4 °C, the supernatant was concentrated and redissolved in 150 μL methanol for LC-ESI-MS/MS detection (UPLC, ExionLCTM AD; MS, QTRAP^®^ System). Based on the standard MetWare database (MWDB), qualitative analysis was conducted according to the retention time of the detected substance, information regarding daughter and parent ion pairs, and secondary spectrum data. Metabolites were quantified using a multi-reaction monitoring model, mainly based on precursor ions, characteristic fragment ions, and the peak area of integrated mass spectrometry.

### Statistical analysis

For differential metabolite analysis, variable importance in projection (VIP) values were extracted from orthogonal partial least squares-discriminant analysis (OPLS-DA) score plot results (which also yielded score plots and permutation plots), generated using the R package MetaboAnalystR. The data was log-transformed (log2) and mean-centered before OPLS-DA. In order to avoid overfitting, a permutation test (200 permutations) was performed.

For clinical data, all values are presented as means ± standard deviation (SD). Two-tailed t-tests were used to determine overall and group pair p-values. The diagnostic metrics of individual and combined biomarker candidates were quantitatively evaluated using receiver operating characteristic (ROC) curve analysis. All statistical analyses were performed using GraphPad Prism 9.0.

## Results

### Baseline clinical characteristics

A total of 78 plasma samples were obtained from 20 healthy controls, 20 patients with T2DM, 18 patients with DKD stage III, and 20 patients with DKD stage IV. Clinical parameters are shown in Table 1, and statistical analysis is shown in Table S1 (Supplementary Material).

**Table 1.** Baseline characteristics by groups, Characteristics and clinical data of the study subjects.

Variables	Groups				Total (*n* = 78)	*p*-value
	HCs (*n* = 20)	T2DM without DKD (*n* = 20)	DKD stage III (*n* = 18)	DKD stage IV (*n* = 20)		
Age (years)	38.9 ± 8.7	54.4 ± 9.5	60.0 ± 7.7	60.7 ± 8.6	53.3 ± 12.3	<0.001
Gender (male, %)	11 (55)	13 (65)	13 (72.2)	13 (65)	50 (64.1)	0.51
Hypertension (*n*, %)	3 (15)	7 (35)	11 (61.1)	16 (80)	37 (47.4)	<0.001
BMI (kg/m^2^)	22.5 ± 2.3	24.2 ± 4.7	25.0 ± 6.3	24.2 ± 2.7	23.9 ± 4.3	0.322
SBP (mmHg)	120.3 ± 14.1	137.1 ± 15.9	143.6 ± 19.0	159.0 ± 29.8	139.9 ± 24.9	<0.001
DBP (mmHg)	70.8 ± 10.0	77.2 ± 10.9	80.4 ± 12.7	80.7 ± 12.0	77.2 ± 12.0	0.076251
UACR (mg/mmol)	–	–	12.4 ± 6.6	191.7 ± 206.3	–	<0.001
BUN (mmol/L)	4.8 ± 0.9	5.9 ± 1.4	6.3 ± 1.4	8.7 ± 2.9	6.4 ± 2.3	<0.001
Creatinine (umol/L)	63.6 ± 14.3	57.2 ± 14.5	65.6 ± 21.0	87.2 ± 43.4	68.4 ± 28.7	<0.01
HbA1c (%)	5.2 ± 0.3	10.4 ± 2.5	9.4 ± 2.1	8.8 ± 1.5	8.4 ± 2.7	<0.001
Hemoglobin (g/L)	132.3 ± 6.9	132.8 ± 13.6	136.4 ± 11.7	120.9 ± 14.6	130.3 ± 13.4	<0.01
Albumin (g/L)	45.6 ± 2.5	42.5 ± 3.45	42.4 ± 3.2	38.7 ± 3.9	42.3 ± 4.1	<0.001
TC (mmol/L)	4.9 ± 0.7	4.6 ± 1.4	4.4 ± 1.2	5.0 ± 1.4	4.7 ± 1.2	0.57352
LDL-c (mmol/L)	3.0 ± 0.6	3.0 ± 1.4	2.7 ± 1.0	3.0 ± 0.9	2.9 ± 1.0	<0.001
HDL-c (mmol/L)	1.3 ± 0.3	1.1 ± 0.3	1.0 ± 0.3	1.2 ± 0.4	1.1 ± 0.3	0.18024
TG (mmol/L)	1.4 ± 1.0	1.6 ± 0.8	1.8 ± 1.1	1.8 ± 1.6	1.6 ± 1.2	<0.001

HCs: Healthy controls; T2DM: Type 2 Diabetes mellitus; DKD: Diabetes kidney disease; BMI: Body Mass Index; SBP: Systolic Blood Pressure; DBP: Diastoli Blood Pressure; UACR: urinaryalbumin⁃to⁃creatinine ratio; HbA1c: Hemoglobin A1c; TC: Total cholesterol; LDL-c: Low density lipoprotein; HDL-c: High density lipoprotein cholesterocholesterin; TG: Triglyceride.

### Profiles and statistical analysis of EV metabolites

**Figure 1.**
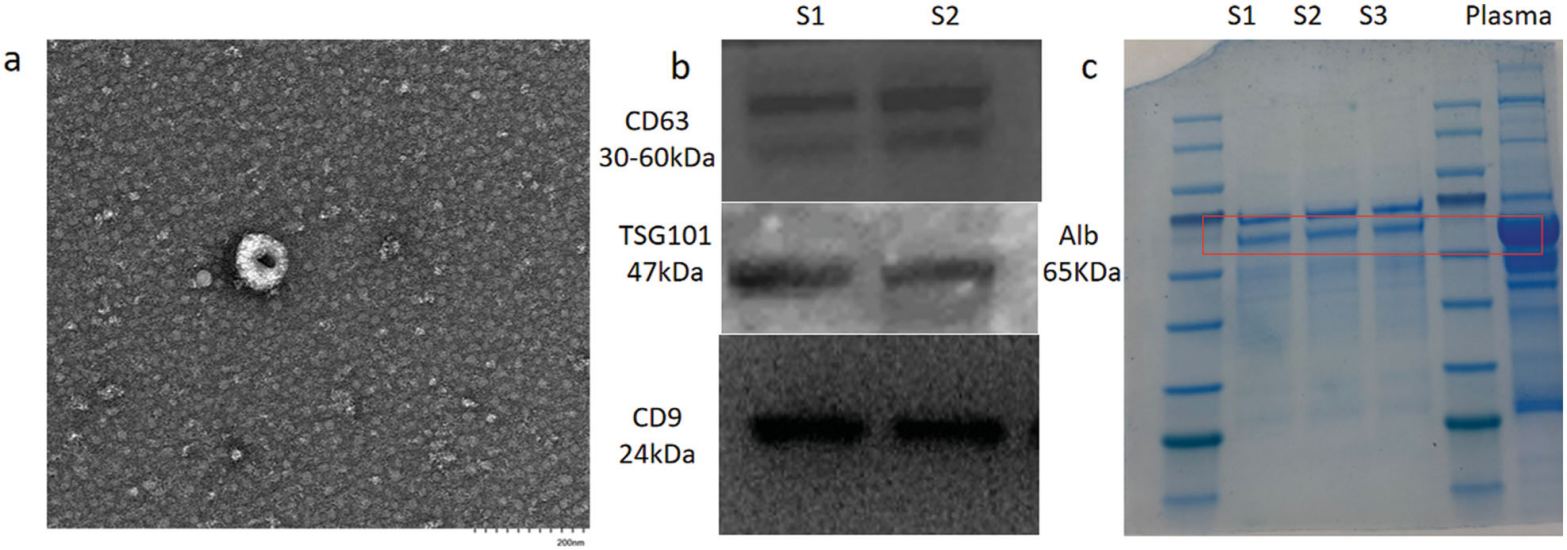
Identification of EVs. (a) Electron microscopic of EVs. (b) western-blot of EVs specific protein. s1, s2 means sample1 and sample 2 respectively. (c) western-blot of nano particles.

**Figure 2.**
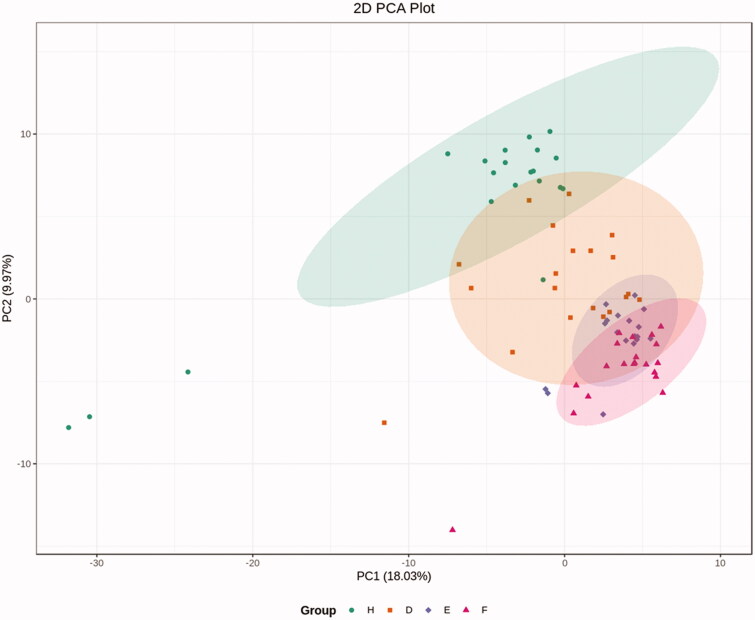
PCA analysis. PCA analysis revealed a significant separation between healthy controls, T2DM patients and DKD patients (including DKD stage III and DKD stage IV). H: healthy controls; D: T2DM without DKD; E: T2DM with DKD stage III; F: T2DM with DKD stage IV.

Following isolation of EVs from healthy controls and the three groups of patients, TEM and western blotting detection of nanoparticles were performed, which showed isolation of EVs with limited non-EV components, while western blotting also showed characteristic protein expression (Figure 1). Based on the LC-ESI-MS/MS platform, extensive targeted metabolic profiling was performed to determine the chemical characteristics of the isolated EVs. As a result, a total of 344 metabolites were identified, including organic acids and their derivatives, amino acids, benzene and its substituted derivatives, alcohols and amines, heterocyclic compounds, nucleosides and their derivatives, aldehydes, ketones, and esters. Principal component analysis (PCA) analysis revealed a significant separation between healthy controls, T2DM patients, and DKD patients (including DKD stage III and DKD stage IV patients), suggesting that metabolic abnormalities are likely to occur in the early stages of DKD (Figure 2).

**Figure 3.**
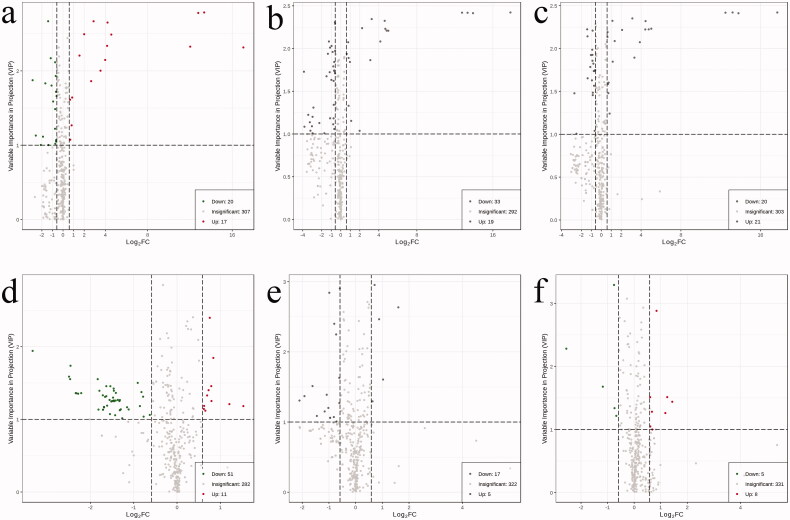
Volcano plot map. EVs differential metabolites that met the criteria (VIP > 1, FC > 1.5, or FC <0.67) were selected between the four groups. Alterations in metabolites between groups are indicated in the lower right corner of each Volcano plot map. (a) Compared with healthy control group, the contents of 20 metabolites in T2DM without DKD group decreased, and the contents of 17 metabolites increased; (b) Compared with healthy control group, the contents of 33 metabolites in DKD stage III group decreased, and the contents of 19 metabolites increased; (c) Compared with healthy control group, the contents of 20 metabolites in DKD stage IV group decreased, and the contents of 21 metabolites increased; (d) Compared with T2DM without DKD group, the contents of 51 metabolites in DKD stage III group decreased, and the contents of 11 metabolites increased; (e) Compared with T2DM without DKD group, the contents of 17 metabolites in DKD stage IV group decreased, and the contents of 5 metabolites increased; (f) Compared with DKD stage III group, the contents of 5 metabolites in DKD stage IV group decreased, and the contents of 8 metabolites increased.

Differential EV metabolites meeting the criteria (VIP >1, FC >1.5, or FC <0.67) were selected for the four groups (healthy controls, T2DM without DKD, DKD Stage III, and DKD Stage IV); these are presented in the form of a volcano map (Figure 3). OPLS-DA modeling was used to discriminate between the four groups, showing good fitness and predictive ability (Supplementary Material, Figure S1–S6).

**Figure 4.**
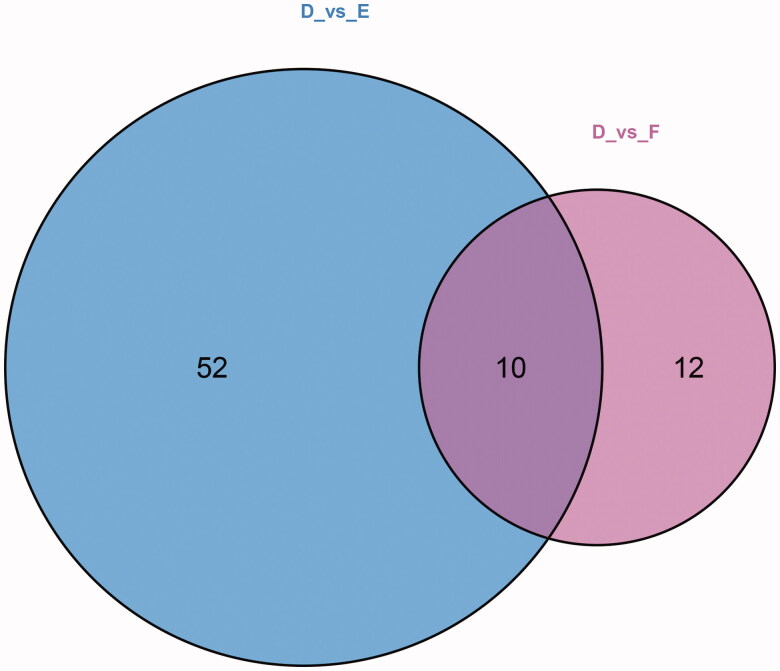
Venn diagram for the metabolites of T2DM without DKD, DKD stages III, and DKD stage IV. D: T2DM without DKD; E: T2DM with DKD stage III; F: T2DM with DKD stage IV. 62 differential metabolites were recognized between T2DM and DKD stages III. 22 differential metabolites were recognized between DKD stages III and DKD stage IV. There were 10 common differential metabolites among the three groups.

The Venn diagram shown in Figure 4 shows 10 differential metabolites that were common to two comparisons, namely, the comparisons between the T2DM without DKD and T2DM with DKD Stage III groups and the T2DM without DKD and T2DM with DKD Stage IV groups. This suggested that these metabolites may be involved in the progression of DKD. The 10 metabolites were uracil, 4-acetamidobutyric acid, ectoine, EPA, sphingosine 1-phosphate (S1P), pyrazine, LPC(O-16:0/0:0), Cer(d18:1/24:1(15Z)), PE(20: 4(5Z, 8Z,11Z,14Z)/P-18:1(11Z)) and LPC(O-18:1/0:0) (Table 2). Among these 10 metabolites, only two increased with disease progression, namely uracil and 4-acetamidobutyric acid. Ectoine levels showed bidirectional alterations, increasing significantly at stage III and decreasing at stage IV, while levels of the remaining seven metabolites were reduced in DKD individuals. Therefore, it was hypothesized that these 10 metabolites might play a role in early DKD and potentially assist in disease staging.

**Table 2.** 10 common metabolite between T2DM and DKD stage III & IV.

Compounds	T2DM_vs_DKD stage III			T2DM_vs_DKD stage IV		
	Fold Change	p value	VIP	Fold Change	p value	VIP
Uracil	1.7295	0.0011	1.4566	1.5512	0.0159	1.2949
4-Acetamidobutyric Acid	1.6888	0.0000	2.3969	3.0293	0.0000	2.6345
Pyrazine	0.4068	0.0214	1.1374	0.4473	0.0424	1.1510
PE(20:4(5Z,8Z,11Z,14Z)/P-18:1(11Z))	0.3759	0.0310	1.3637	0.5675	0.1875	1.0709
Cer(d18:1/24:1(15Z))	0.3748	0.0232	1.2582	0.4720	0.0632	1.3891
Ectoine	2.8904	0.0384	1.1821	0.4924	0.0041	1.2010
EPA	0.3226	0.0229	1.4561	0.3651	0.0304	1.0865
Sphingosine 1-phosphate	0.3442	0.0239	1.4557	0.3263	0.0203	1.5121
PC(O-16:0/0:0)	0.1777	0.0128	1.5872	0.2326	0.0207	1.3062
LPC(O-18:1/0:0)	0.1820	0.0104	1.7373	0.2647	0.0215	1.3691

**Figure 5.**
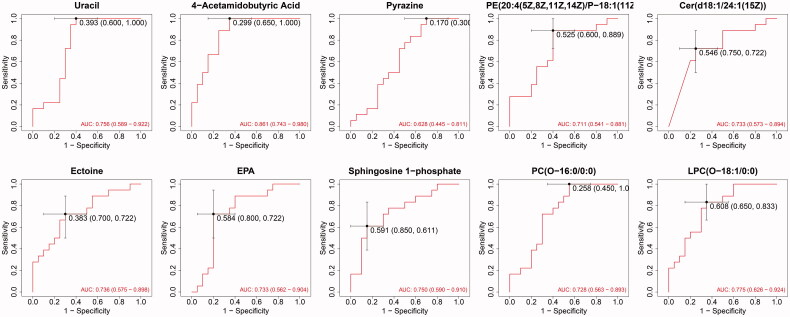
ROC curves of 10 common differential metabolites. The AUC value: uracil: 0.756; 4-acetamidobutyric acid: 0.861; ectoine: 0.736; EPA: 0.733; sphingosine 1-phosphate: 0.750; pyrazine: 0.628; LPC(O-16:0/0:0): 0.728; Cer (d18:1/24:1(15Z)): 0.733; PE (20: 4(5Z, 8Z,11Z,14Z)/P-18:1(11Z)): 0.711; LPC (O-18:1/0:0):0.775.

**Figure 6.**
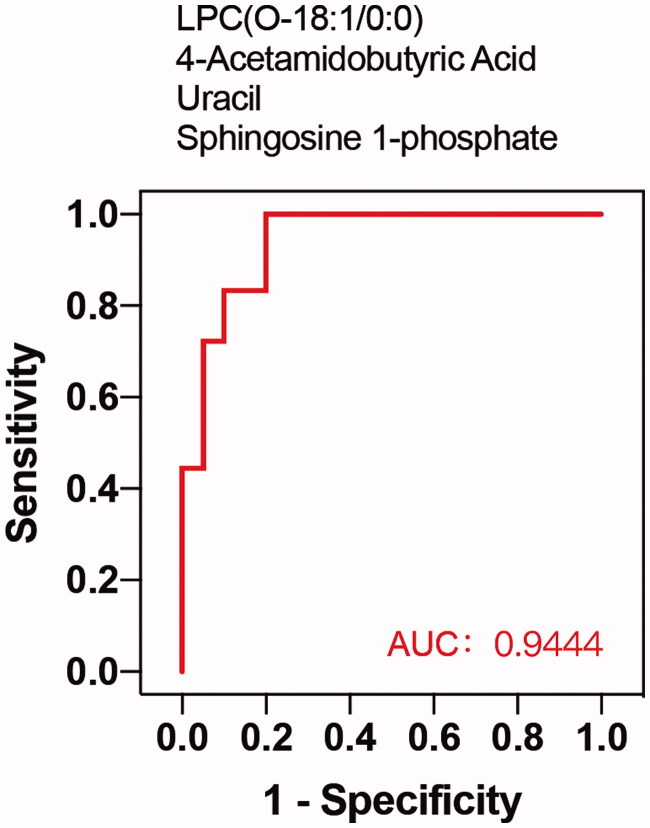
ROC curve of combination of 4 metabolites. The combination of 4 candidate metabolites showed an excellent prediction for DKD with 0.944 of AUC.

**Figure 7.**
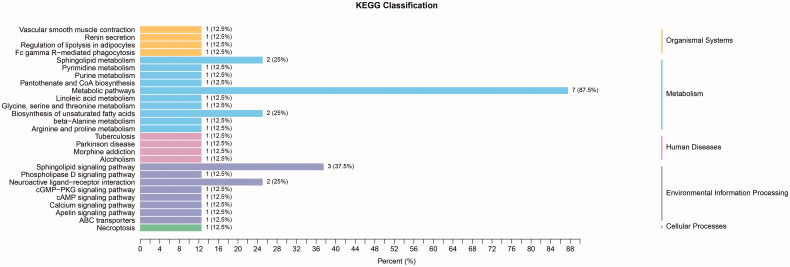
KEGG classification of T2DM without DKD vs DKD stage III. 3 pathways were significantly enriched, namely metabolic pathways, sphingolipid signaling pathway, and biosynthesis of unsaturated fatty acid.

Among the metabolites significantly increased during diabetes development, four showed diagnostic potential, with the ability to distinguish T2DM without DKD from DKD (Figure 5), as determined by area under the curve (AUC). These were uracil (AUC 0.756), LPC(O-18:1/0:0) (AUC 0.775), S1P (AUC 0.750), and 4-acetamidobutyric acid (AUC 0.861). In addition, ROC analysis of the four selected metabolites combined revealed excellent predictive capability for early DKD (Figure 6). Furthermore, KEGG pathway analysis (Figure7) showed significant enrichment of three pathways, namely metabolic pathways, the sphingolipid signaling pathway, and biosynthesis of unsaturated fatty acids.

## Discussion

DKD is a major cause of chronic kidney disease (CKD) and ESKD [23], with irreversible kidney impairments arising when patients have obvious manifestations of disease [24]. According to current diagnostic criteria, when patient diagnoses are confirmed, the patients have already reached stage III. Hence, early identification and intervention are of great importance in order to reduce and delay the progression of DKD to ESKD [25]. However, early diagnosis of DKD has always been problematic for clinicians. Therefore, increasing attention is being paid to early diagnosis methods.

Because T2DM is a metabolic disease, the metabolic molecules carried by EVs in DKD patients have become the focus of increased attention. Furthermore, changes in the composition of EV metabolites may be closely related to the pathological state of DKD. Plasma contains both free metabolites as well as metabolites carried by EVs. When detecting plasma metabolites, all metabolites are generally included. However, studies focused on metabolites in EVs provide new perspectives and more accurate conclusions. This study aimed to explore potential biomarkers for the prediction and early diagnosis of DKD by analyzing EV metabolites. Ten metabolites were selected, four of which showed potential for predicting DKD. In addition, a model constructed to combine the four metabolites showed excellent predictive ability.

As a GABA derivative and an important intermediate in arginine and proline metabolism, 4-acetamidobutyric acid is an essential component in amino acid metabolism. In a mouse model of DKD, Bai Linnan et al. used situ metabolomics to show perturbation in arginine and proline metabolism [26]. An earlier study showed that plasma levels of amino acids such as proline, ornithine, and citrulline, were significantly elevated in uremic patients before dialysis, compared with controls, indicating abnormalities in amino acid metabolism, especially proline metabolism [27]. Additionally, a previous study showing that 4-acetamidobutyric acid may have a potential role in proliferative diabetic retinopathy [28] suggests that 4-acetamidobutyric acid may be involved in the progression of DKD, as DKD and diabetic retinopathy are strongly associated. This study found that the level of 4-acetamidobutyric acid increased gradually with disease progression; therefore, this metabolite has potential as a biomarker of DKD.

Another metabolite, S1P, was discovered as the end product of sphingolipid metabolism in the 1960s [29]. It was later found to have unique physicochemical properties that made it versatile during development as well as in various physiological and pathological contexts [30]. The S1P-S1PR1 signaling pathway maintains endothelial barrier function to limit plasma and leukocyte extravasation [31,32], while multiple studies have shown that endothelial dysfunction plays a pivotal role in the early pathogenesis and progression of DKD [33,34]. Notably, S1P signaling outcomes are dependent on downstream effectors of selective G-protein-coupled S1P receptors, which are differentially expressed in diabetic kidneys [35]. Another study verified increased S1P-mediated inflammation and fibrosis in tubular injury in diabetic nephropathy [36]. S1P is primarily produced by erythrocytes, and its concentration in blood and tissue is considered a biomarker for some diseases [37]. This study found decreased levels of S1P in DKD patients, consistent with the finding from KEGG pathway analysis, suggesting impaired S1P-S1PR1 signaling in DKD.

Another sphingomyelin, Cer(d18:1/24:1(15Z)) was identified in our study. Four sphingomyelin metabolites in serum have been shown to correlate with UACR [38]. In the pathological process of DKD, increased activity of sphingomyelin choline synthase leads to the accumulation of sphingomyelin choline in podocytes, while inhibition of sphingomyelin choline conversion in animal studies can alleviate podocyte injury and reduce proteinuria [39,40]. Recent studies have proposed the pathological mechanism of ceramide-mediated kidney disease [41]. The two sphingolipids, S1P and Cer (d18:1/24:1(15Z)), identified in this study were highly correlated with DKD and, thus, may be useful as early biomarkers of DKD.

Pseudouridine, a derivant of uracil, was reported to increase in patients with diabetes who progressed to ESKD, compared with those who did not, suggesting that uracil may be directly or indirectly associated with pathological changes in the kidney [42]. Pseudouridine levels in plasma increased as urinary excretion decreased, accumulating significantly in the uremic state, consistent with the results of increased uracil content in EVs.

EPA, an omega-3 fatty acid, has been shown to prevent inflammation and metabolic disorders, while lack of ω-3-polyunsaturated fatty acids has been shown to aggravate diabetes retinopathy. In addition, deficiency of ω-3-polyunsaturated fatty acids reportedly down-regulated the expression of E-cadherin and desmoglein—key molecules for the adequate sealing of epithelial-conjunctive barriers—in squamous skin cells [43]. This may be related to the mechanism of glomerular injury in DKD. In this study, EPA carried in the plasma exosomes of DKD patients showed a downward trend correlated with disease progression, suggesting its potential to predict disease progression [44].

Several abnormal lipid metabolites were also identified in this study, including PE(20:4(5Z,8Z,11Z,14Z)/P-18:1(11Z)), PC(O-16:0/0:0), and LPC(O-18:1/0:0). Unsurprisingly, decreased levels of these glycerol phospholipids strongly hint at the impairment of the glycerol phospholipid biosynthesis pathway. These abnormal lipid metabolites have been associated with disease in some cancer studies; for example, serum levels of LPC(16:0/0:0) were decreased in kidney cancer patients [45]. Although there have been few publications discussing a direct association between unsaturated fatty acids and DKD, low linolenic and linoleic acid intake are associated with CKD in T2DM patients, suggesting an insufficiency of glycerol phospholipid in DKD patients [46]. In our study, KEGG pathway analysis showed that the unsaturated fatty acids biosynthesis pathway was significantly affected, similar to the metabolic reprogramming observed in a mouse model of DKD [26].

Pyrazine (an isomer of pyrimidine) is a member of the 1,4-diazine group of organic compounds, which constitute an important class of heterocyclic molecules. Pyrazine derivatives exert numerous noteworthy pharmacological effects, including antimycobacterial, antibacterial, antifungal, antidiabetic, diuretic, anticancer, antiviral, hypnotic, and analgesic activities [47]. However, the role of pyrazine in DKD, especially in EV metabolomics, remains to be determined.

Unlike the above nine metabolites, ectoine levels were increased in DKD stage III samples but reduced in DKD stage IV samples. Ectoine has been proven to have enzyme-stabilizing and anti-inflammatory properties, exerting protective effects in human skin as well as inhibitory effects in neurodegenerative diseases, while therapeutic potential has been demonstrated in human and veterinary medicine [48]. The change in ectoine content observed during DKD progression suggests that the increase occurring in early DKD may serve as a compensatory mechanism.

ROC curve analysis was used to evaluate the diagnostic performance of these potential biomarker candidates, while multivariate logistic regression analysis was performed to establish ROC curves of biomarker combinations. The results showed that 4-acetamidobutyric acid can be considered a potential biomarker for early diagnosis, with an AUC value of 0.861, while S1P, LPC(O-18:1/0:0), and uracil are also acceptable, with AUC values of 0.750, 0.775 and 0.756, respectively. Considering the effects of individual factors on single metabolites, we analyzed the ROC curve of the combination of these four candidate metabolites. Interestingly, this combination showed an excellent predictive capacity for DKD, with an AUC of 0.944. Thus, although the pathways of the four metabolites do not interact, we believe that combining metabolites produced by different pathways may lead to enhanced predictive effects.

Although several significant metabolites and metabolic pathways have been identified in this study, the number of samples analyzed was a limitation. Further studies with increased numbers of patient samples are required for verification. In addition, although the combination of biomarkers showed striking results, further verification is required.

## Conclusion

In conclusion, the analysis of plasma EVs in this study revealed characteristic alterations in the metabolomic patterns of T2DM patients with and without DKD. Three pathways were significantly affected, including metabolic pathways, the sphingolipid signaling pathway, and biosynthesis of unsaturated fatty acids. The metabolites 4-acetamidobutyric acid, S1P, LPC(O-18:1/0:0), and uracil were identified as suitable biomarkers for the early diagnosis of DKD. Furthermore, a combined model using these four metabolites showed excellent potential for the early diagnosis of this condition.

## Supplementary Material

Supplemental Material
