# Supplementary material for: Extracellular vesicles metabolic changes reveals plasma signature in stage-dependent diabetic kidney disease
Source: Ren Fail. 2022 Nov 11;44(1):1850–9. doi: 10.1080/0886022X.2022.2118067 (PMC9662026; doi:10.1080/0886022X.2022.2118067)
Supplement: Supplemental Material [file IRNF_A_2118067_SM6100.pdf]

Table 1. The specific group comparison P-value of Baseline characteristics

Variables	Groups			
	HCs（n=20）	DM without DKD(n=20)	DKD stage Ⅲ（n=18）	DKD stage Ⅳ（n=20）
Age（years）	-	0.000005	2.7518E-09	1.44E-09
vs DM without DKD		-	0.062355	0.039923
vs DKD stage III			-	0.813111
Gender（male，%）	-	2.14E-01	0.094354	0.213606
		-	0.643398	1
			−	0.643398
Hypertension(n,%)	-	0.080194	0.000661	0.000005
		-	0.105579	0.008919
			-	0.364903
BMI（kg/m2)	-	0.160141	0.12548	0.051962
		-	0.693082	0.964245
			-	0.624617
SBP（mmHg）	-	0.001126	0.000133	0.000008
		-	0.274411	0.007601
			-	0.076273
DBP（mmHg）	-	0.060829	0.014123	0.00762
		-	0.421667	0.351121
			-	0.940025
UACR(mg/mmol)	-	-	-	-
		-	-	-
			-	0.000984
BUN(mmol/L)	-	0.007113	0.000348	0.000002
		-	0.342477	0.000474
			-	0.004161
Creatinine(umol/L)	-	0.186013	0.732553	0.030696
		-	0.164952	0.006873
			-	0.070811
HbA1c（%）	-	8.42E-11	1.65E-10	4.02E-13
		-	0.20503	0.022113
			-	0.316487
Hemoglobin(g/L)	-	0.886898	0.206331	0.003819
		-	0.411828	0.013343
			-	0.001618
Albumin(g/L)	-	0.002883	0.002069	1.03E-07
		-	0.955912	0.003113
			-	0.003881
TC(mmol/L)	-	0.4479	0.116209	0.614487

		-	0.577503	0.324201
			-	0.120646
LDL-c(mmol/L)	-	0.990808	0.322806	0.847252
		-	0.524482	0.89617
			-	0.327613
HDL-c(mmol/L)	-	0.038152	0.012196	0.292579
		-	0.654381	0.507798
			-	0.309708
TG(mmol/L)	-	0.672177	0.347226	0.407335
		-	0.53115	0.575988
			-	0.952418

BMI:Body Mass Index；SBP:Systolic Blood Pressure；DBP:Diastoli Blood Pressure；UACR:urinary albumin-to-creatinine ratio；HbA1c:Hemoglobin A1c；TC:Total cholesterol；LDL-c:Low density lipoprotein ；HDL-c:High density lipoprotein cholestero cholesterin；TG:Triglyceride.
